# Optimized decision algorithm for the microbiological diagnosis of osteoarticular infections in adults using synovial fluid samples: a prospective study in two French hospitals including 423 samples of synovial fluid

**DOI:** 10.5194/jbji-9-37-2024

**Published:** 2024-02-06

**Authors:** Céline Dupieux, Ghislaine Descours, Paul Verhoeven, Florence Grattard, Yvonne Benito, François Vandenesch, Céline Cazorla, Tristan Ferry, Sébastien Lustig, Bertrand Boyer, Sandrine Boisset, Anne Carricajo, Frédéric Laurent, / PIRLA investigator group

**Affiliations:** 1 Laboratoire de Bactériologie, Institut des Agents Infectieux, Hôpital de la Croix-Rousse, Hospices Civils de Lyon, Lyon, 69004, France; 2 Laboratoire de Bactériologie, CHU de Saint-Étienne, Saint-Étienne, 42055, France; 3 Service de Maladies Infectieuses, CHU de Saint-Étienne, Saint-Étienne, 42055, France; 4 Service de Maladies Infectieuses, Hôpital de la Croix-Rousse, Hospices Civils de Lyon, Lyon, 69004, France; 5 Service de Chirurgie Orthopédique, Hôpital de la Croix-Rousse, Hospices Civils de Lyon, Lyon, 69004, France; 6 Service de Chirurgie Orthopédique, CHU de Saint-Étienne, Saint-Étienne, 42055, France; ➕ A full list of authors appears at the end of the paper

## Abstract

No consensus exists about the techniques to use for microbiological diagnosis of bone and joint infections (BJIs). The objective herein was to define an algorithm to optimize BJI diagnosis in adults using various bacteriological methods on synovial fluid samples. This prospective multi-center study included 423 synovial fluids collected from adult patients with suspected BJIs. Culture (using five solid media, an enrichment broth, and blood culture bottles), universal 16S rRNA PCR followed by Sanger sequencing, and seven specific bacterial PCRs were systematically performed. Combinations of methods were compared to arrive at the optimized algorithm. Among 423 synovial fluids, 242 infections were diagnosed (57.2 %): 213 mono- and 29 poly-microbial for a total of 284 bacteria (staphylococci at 54.6 %, streptococci–enterococci at 16.5 %, Gram-negative bacilli at 15.5 %, anaerobic species at 8.8 %). Comparing culture techniques, blood culture bottles had the highest sensitivity (67.6 % for pediatric and 63.9 % for anaerobic bottles) but are not sufficient alone and require being combined with solid media. The 16S rDNA PCR detected only 52.3 % of the bacteria, whereas specific PCRs had a higher sensitivity (*Staphylococcus* spp. at 66.2 %, *S. aureus* at 85.2 %, *Streptococcus* spp. at 91.2 %). Based on these results, an algorithm was proposed associating three solid media; inoculation into blood culture bottles; and 16S, *Staphylococcus* spp., and *Streptococcus* spp. PCRs, which would have detected 90.5 % of bacteria in the present cohort versus 79.2 % using all culture techniques on synovial fluid. This prospective study shows that a combination of culture and molecular methods on synovial fluids allows the optimization of bacterial detection.

## 1 Introduction

A precise microbiological diagnosis is crucial in bone and joint infections (BJIs) to enhance the chance of successful treatment (Lew and Waldvogel, 2004). This consists of identifying the causative microorganisms and determining the appropriate antibiotic therapy by performing antimicrobial susceptibility testing (AST) when possible (Tande and Patel, 2014). Nevertheless, there is no consensus about the techniques that should be used to optimize the detection of pathogens responsible for BJIs.

Culture can be considered to be the gold standard for BJI diagnosis; it allows AST to be performed (Osmon et al., 2013). Based on literature, culture should be performed from five osteoarticular samples, prolonged to 14 d, in aerobic and anaerobic atmospheres using four to six media, combining agar plates and liquid broths with early and/or late reading (Schäfer et al., 2008; Larsen et al., 2012). The multiplicity of media and the long duration of incubation makes the microbiology monitoring of osteoarticular samples very complex and expensive (Peel et al., 2017). However, regarding synovial fluids (SFs), no exhaustive study has compared the performance and the value of the various culture media used or evaluated the possibility of improving the performance or simplifying this process, in particular by combining classical culture with new techniques (Hughes et al., 2011; Bémer et al., 2016). For instance, inoculation of SF or crushed osteoarticular biopsies in blood culture (BC) bottles seems to be an interesting approach and could replace liquid broths (Font-Vizcarra et al., 2010; Minassian et al., 2014; Peel et al., 2016). Another approach would be the combination of culture and molecular methods. Molecular assays can be used on osteoarticular samples to overcome the poor performance of culture in the case of antimicrobial chemotherapy before samples or in the case of fastidious or non-cultivable microorganisms (Levy et al., 2013). For this, two approaches are possible; a broad-range (universal) PCR targeting 16S ribosomal DNA followed by sequencing is useful to identify the causative microorganism irrespective of the bacterial species involved, but methods using Sanger sequencing are classically less sensitive than culture (Bémer et al., 2014; Lallemand et al., 2016; Jacquier et al., 2019), while specific PCR assays targeting particular bacteria are more sensitive, but this restricts the diagnosis to the sought species (Chometon et al., 2007; Levy et al., 2013).

In this context, studies are needed to evaluate the performance of each available technique and to determine a decisional algorithm in order to improve the diagnosis of BJIs but also to reduce the costs and to standardize and simplify the procedures. Herein we report the results of a prospective multi-center study, the objective of which was to provide an algorithm of the culture media to use and the situations in which molecular assays are useful. For this, we compared the performance of a large panel of bacteriological techniques (classical culture on different media, inoculation of SF in BC bottles, and systematic broad-range and specific PCR assays).

## 2 Patients and methods

### 2.1 Study design and data collection

The PIRLA study (Protocole Inter-Régional Liquide Articulaire) was designed as a multi-center, prospective, observational study. Consecutive adult patients with SF punctures and clinical signs suggesting acute or chronic BJI in two French university hospitals (Hospices Civils de Lyon and Centre Hospitalier Universitaire de Saint-Étienne) between April 2011 and April 2014 were included. Suspicion of BJI was based on the presence of evocative symptoms (pain, fever, functional impotence, sinus tract, or joint effusion). Case report forms (CRFs) were used to collect clinical data for each patient: patient characteristics, surgical and infectious history, presentation and site of infection, presence or absence of foreign material (prosthesis, cements, osteosynthesis), antibiotic treatment in the 15 d before and after SF collection, and microbiological results of other osteoarticular samples collected before or at the same time as the SF.

### 2.2 Microbiological methods

The SF sample was collected under sterile conditions, either in operating rooms or at the patient's bedside, and placed into a sterile vial and an ethylenediaminetetraacetic acid (EDTA) tube. SF was also inoculated into two BC bottles (pediatric – PED and anaerobic – ANA) directly by the clinicians (Bactec™ Peds Plus/F and Bactec™ Lytic/10 Anaerobic/F from Becton Dickinson, Franklin Lakes, NJ, US, in Saint-Étienne; BacT/ALERT™ PF and BacT/ALERT™ FN from bioMérieux, La-Balme-les-Grottes, France, in Lyon; inoculated volumes were not controlled, and suggested volumes were at least 5 mL per bottle). Samples were shipped to the bacteriology laboratory of the respective hospitals at room temperature within 4 h after collection.

BC bottles containing SF were incubated for 15 d. If found to be positive, a Gram staining and a subculture were performed on various plates according to the results of Gram staining and the aerobic or anaerobic nature of the positive bottle. Cultures and Gram coloration of SF samples in sterile vials were performed following a standardized protocol. Approximately 50 µL of SF were inoculated on a Columbia sheep blood agar plate (BA), two PolyVitex chocolate agar plates (CA), and two blood agar plates for anaerobic incubation (BAana) and in a Schaedler anaerobic liquid broth (SCH broth; all media from bioMérieux). All media were incubated at 36 ^∘>^C, with BA in an aerobic atmosphere, CA plates in 5 % CO_2_, and BAana plates in an anaerobic atmosphere. BA and the first CA plates were incubated for 48 h and read at 24 and 48 h; the first BAana plate was incubated for 72 h; the second CA and BAana plates were incubated for 10 d. SCH broth was observed every day and sub-cultured, as soon as it became cloudy, on two blood agar plates incubated in 5 % CO_2_ and in an anaerobic atmosphere; if not cloudy, broth was systematically sub-cultured on the 10th day on CA and BAana plates incubated for 5 d in 5 % CO_2_ and anaerobic atmosphere, respectively. Isolated bacteria were identified according to standard laboratory procedures: VITEK 2 system or VITEK MS MALDI-TOF (bioMérieux) in Lyon and MALDI Biotyper (Bruker Daltonics, Bremen, Germany) in Saint-Étienne.

### 2.3 Molecular methods

SF samples were systematically tested using various bacterial PCRs in parallel with cultures: 16S rDNA PCR followed by Sanger sequencing and seven specific PCRs targeting (i) *Staphylococcus* spp., (ii) *S. aureus*, (iii) *Streptococcus* spp., (iv) *S. pneumoniae*, (v) *Kingella kingae*, (vi) *Borrelia burgdorferi*, and (vii) *Cutibacterium acnes* (Table A1 in the Appendix; in-house PCR assays except *Borrelia*).

SF samples were pre-treated with proteinase K for 3 h at 63 ^∘^C. DNA extraction was performed from 400 µL of SF supernatant using the MagNA Pure Compact System (Roche Diagnostics) in the Lyon laboratory and the easyMAG system (bioMérieux) in the Saint-Étienne laboratory, as previously described (Chometon et al., 2007). In each PCR series, we performed a negative control and an internal control by PCR targeting human beta-globin to check the absence of PCR inhibitors and validate the extraction efficiency.

Regarding 16S rDNA, *Staphylococcus* spp.  and *Streptococcus* spp. PCR assays, if positive, amplification products were secondarily sequenced by means of the Sanger method, and the sequences were compared to those of the Bioinformatics Bacteria Identification (BIBI) database. Sequence similarities of >98 % and >96 % were used to define the identification at the species level and at the genus level, respectively. Uninterpretable results were considered to be negative because they were non-contributive with regard to diagnosis.

### 2.4 Analysis and interpretation of results

For each SF tested, the patient was classified as infected or not infected according to the French 2022 national recommendations (Référentiel en microbiologie médicale, REMIC; Sect. S1 in the Supplement). Each microorganism detected by either culture and/or PCR was defined as responsible for infection or contamination according to its pathogenicity, the microbiological results of other bone samples, and the clinician's decision to treat or not treat this bacterium. The sensitivity of each culture medium used and each PCR performed was calculated overall and then by species, genus, or family of bacteria. The specificity of PCR assays was also determined. For polymicrobial infections, sensitivity was calculated individually for each bacterium as not all bacteria were always detected by all techniques used.

### 2.5 Statistical analysis and construction of the diagnostic algorithm

The comparative performance of the different media or PCRs was assessed in terms of detection rate using the Chi-squared test. The significance threshold was set at 0.05. To determine the algorithm allowing us to optimize bacterial detection, the detection rate of several combinations of the different culture media and PCRs used were compared with those obtained using all the techniques combined.

## 3 Results

### 3.1 Population characteristics and sample collection

Among the 480 SF from adult patients identified, 57 were excluded because of a lack of written consent and/or a missing CRF; a total of 423 samples from 332 patients were included. There were 256 patients who had only one SF included in the study, while 76 had several SFs collected at different time points (two, n=64 ; three, n=9 ; four, n=3 ). Each SF was analyzed individually, even if punctured from the same patient. Of the 423 samples included, 218 were collected from male patients (51.5 %); the age of patients ranged from 19 to 98 years (median age: 69 years, interquartile range: 56–78); and the most frequent sites of puncture were the knee ( n=226 , 53.4 %) and the hip ( n=136 , 32.2 %). For 276 samples (65.2 %) the patient had or have had prosthetic material at the punctured joint, and for 132 samples (31.2 %) the patient received antibiotics in the 2 weeks before SF collection (Table 1).

**Table 1:** Characteristics of the cohort and collection of samples analyzed.

	Total of samples	Synovial fluids from native joint	Synovial fluids from non-native joint^a^
	n=423	n=136	n=287
Sex, male number/sex ratio	218/1.06	82/1.52	136/0.90
Age at joint puncture, years			
Median	69	60	71
Range	19–98	19–98	31–96
Interquartile range	56–78	45–73	63–78
Location of joint puncture			
Knee	226	68	158
Hip	136	14	122
Vertebral column	22	22	0
Shoulder	16	12	4
Ankle	12	12	0
Elbow	1	1	0
Others (hands or feet joints, osteitis)	10	7	3
Patients having received antibiotics in the 2 weeks before sample, n (%)	132 (31.2)	34 (25.0)	98 (34.1)
Confirmed BJI, n (%)	242 (57.2)	73 (53.7)	169 (58.9)
Acute infection^b^	111	51	60
Chronic infection^b^	130	22	108

^a^ Non-native joints correspond to 276 prosthetic joints and 11 joints with other orthopaedic material. ^b^ In one case, no information was available on whether the infection was acute or chronic (differentiation between acute and chronic infection defined as clinical evolution of < or >4 weeks).

### 3.2 Overall bacteriological results of synovial fluids analyzed using the combination of all the techniques used

After analysis of bacteriological results and clinical decision, an infection was confirmed in 242 cases: 213 (88.0 %) were monomicrobial, and 29 (12.0 %) were polymicrobial, for a total of 284 bacteria.

**Figure 1:**
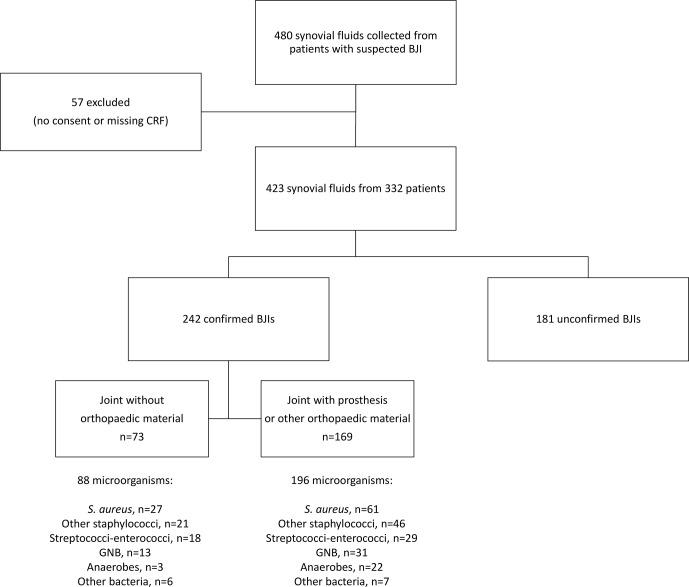
Flowchart of enrolled patients and microbiological results of confirmed BJIs. BJI indicates bone and joint infection, CRF indicates case report form, and GNB indicates Gram-negative bacilli.

Among the monomicrobial infections, staphylococci were identified in 131 cases (61.5 %) – of which 83 were *S. aureus* (38.9 %), streptococci and enterococci were identified in 30 cases (14.1 %), Gram-negative bacilli were identified in 29 cases (13.6 %), anaerobes were identified in 13 cases (6.1 %), and other bacteria were identified in 10 cases (4.7 %). The 29 polymicrobial cases involved two bacterial species in 22 cases, three species in 4 cases, four species in 1 case, five in 1 case, and six in 1 case. Of these 71 bacteria present in polymicrobial infections, 24 were staphylococci (33.8 %), 17 were streptococci or enterococci (23.9 %), 12 were anaerobes (16.9 %), 15 were Gram-negative bacilli (21.1 %), and 3 were other bacteria (4.2 %). The results according to the presence or absence of orthopaedic material are presented in Fig. 1.

Of these 242 cases of infection, SF analysis using all techniques detected 266 (93.7 %) out of the 284 bacteria and was completely negative in 9 (3.7 %) cases. The 18 bacteria responsible for BJIs not detected in SF were detected by culture of intraoperative tissues or by specific PCR not performed in the study protocol. A total of 58 other bacteria in 53 samples were detected in SF but were, in the end, considered to be contaminants (Table S1).

**Table 2:** Sensitivity of each culture medium and incubation time for the detection of bacteria responsible for 242 bone and joint infections in synovial fluid.

		Sensitivity (%)							
		BA aero.	CA CO_2_	BA ana.	CA CO_2_	BA ana.	SCH broth	BC	BC
		(2 d)	(2 d)	(3 d)	(10 d)	(10 d)	(10 d)	ANA	PED
Anaerobic species, n=25		0	0	28.0	0.0	40.0	40.0	48.0	12.0
	incl. *Cutibacterium acnes*, n=12	0	0	16.7	0.0	33.3	25.0	41.7	16.7
*Streptococcus-Enterococcus* spp., n=47		38.3	36.2	38.3	36.2	40.4	40.4	54.3	54.3
	incl. *Enterococcus* spp., n=12	41.7	41.7	41.7	41.7	50.0	58.3	66.7	58.3
	incl. *Streptococcus* spp., n=35	37.1	34.3	37.1	34.3	37.1	34.3	50.0	52.9
	incl. *S. pneumoniae*, n=2	50.0	50.0	50.0	50.0	50.0	50.0	50.0	50.0
*Staphylococcus* spp., n=155		54.2	54.2	51.3	53.9	54.5	58.7	73.7	79.7
	incl. *Staphylococcus aureus*, n=88	76.1	77.3	75.9	77.3	75.0	77.3	75.6	85.2
	incl. non-*aureus* staphylococci, n=67	25.4	23.9	18.5	22.7	27.3	34.3	71.2	72.3
Gram-negative bacilli, n=44		58.1	46.5	31.7	45.2	38.1	60.5	63.6	81.8
Other bacteria, n=13		7.7	30.8	0.0	30.8	0.0	0.0	15.4	30.8
All bacterial species, n=284		45.2	44.2	41.7	43.8	45.9	51.8	63.9	67.6
	incl. monomicrobial, n=213	48.6	46.7	45.9	47.1	49.0	54.5	64.6	71.9
	incl. polymicrobial, n=71	35.2	36.6	29.0	33.8	36.6	43.7	62.0	54,9

BA indicates blood agar, aero. indicates aerobic atmosphere, CA indicates chocolate agar, ana. indicates anaerobic atmosphere, SCH indicates Schaedler, BC ANA indicates anaerobic blood culture bottle, BC PED indicates pediatric blood culture bottle, and incl. indicates including.

### 3.3 Detection of bacteria in culture

Overall, the sensitivity of the various media inoculated ranged from 41.7 % to 51.8 % for culture media and from 63.9 % to 67.6 % for BC bottles (Table 2). More precisely, the detection rate of the 284 microorganisms (listed in Table B1) was 45.2 % on the BA plate and 44.2 % on the CA plate at 2 d of incubation, 41.7 % on the BAana plate at 3 d, and 43.8 % on the CA plate and 45.9 % on the BAana plate at 10 d; it was 51.8 % in SCH broth and 63.9 % and 67.6 % in the ANA and PED BC bottles. For the 25 anaerobes, sensitivity was 0 % on BA and CA plates at 2 d of incubation, 28.0 % on the BAana plate at 3 d, and 40.0 % on the BAana plate at 10 d; it was also 40.0 % in SCH broth and 48.0 % in the ANA BC bottle. This demonstrated the value of the BC bottles for both aerobic and anaerobic species since they have a similar or greater sensitivity compared to classical culture media (agar plates and Schaedler broth). For the 212 microorganisms isolated in BC bottles, 74.0 % were positive in both bottles, and 26.0 % were positive in only one (15.6 % in the PED and 10.4 % in the ANA).

The positivity rates for combinations of different culture media associated with BC bottles were calculated. A combination of three culture media (BA and CA plates for 48 h and BAana plate for 10 d) gave results which were not significantly different ( p=0.24 ) from those obtained with the six culture media (respectively, 52.8 % vs. 58.1 % positivity rate). When adding BC bottles to these three media, 77.1 % of microorganisms were detected compared to 79.2 % with all culture media tested and BC bottles ( p=0.61 ; Table 3).

**Table 3:** Sensitivity of various culture and PCR method combinations for the detection of bacteria responsible for 242 bone and joint infections in synovial fluid.

		Detection rate (% )								
		3 d^a^ culture	All 3 and 10 d	2 d culture media and	BC bottles	All 3 and 10 d	2 d culture media and	All 3 and 10 d culture	2 d culture media	2 d culture media
		media only	culture media^b^	10 d BA ana^c^ only	only	culture media^b^ BC	10 d BA ana^c^ BC	media^b^ BC + 16S PCR	and 10 d BA ana^c^ +	and 10 d BA ana^c^ +
									BC + 16S PCR	BC + 16S PCR
										+ specific PCR
Anaerobic species, n=25		28.0	48.0	40.0	52.0	64.0	52.0	68.0	60.0	64.0
	incl. *Cutibacterium acnes*, n=12	16.7	33.3	33.3	41.7	50.0	50.0	58.3	58.3	66.7
*Streptococcus-Enterococcus* spp., n=47		40.4	44.7	42.6	57.4	63.8	61.7	80.9	80.9	93.6
	incl. *Enterococcus* spp., n=12	41.7	58.3	50.0	66.7	75.0	66.7	83.3	83.3	83.3
	incl. *Streptococcus* spp., n=35	40.0	40.0	40.0	55.9	60.0	60.0	80.0	80.0	97.1
*Staphylococcus* spp., n=155		54.8	63.2	57.4	85.1	88.4	87.1	91.6	90.3	94.8
	incl. *Staphylococcus aureus*, n=88	77.3	78.4	77.3	87.5	89.8	88.6	94.3	93.2	96.6
	incl. non-*aureus* staphylococci, n=67	25.4	43.3	31.3	81.8	86.6	85.1	88.1	86.6	92.5
Gram-negative bacilli, n=44		59.1	65.9	61.4	84.1	84.1	84.1	97.7	97.7	97.7
Other bacteria, n=13		30.8	38.5	30.8	30.8	38.5	38.5	61.5	61.5	84.6
All bacterial species, n=284		49.6	58.1	52.8	74.9	79.2	77.1	87.3	85.9	91.9
	incl. monomicrobial, n=213	51.6	57.3	53.5	76.9	77.5	77.0	88.3	87.8	94.8
	incl. polymicrobial, n=71	43.7	60.6	50.7	69.0	84.5	74.6	84.5	80.3	83.1

^a^ BA and CA incubated 2 d and BAana incubated for 3 d. ^b^ All gelose agar media and SCH broth incubated 2, 3, and 10 d. ^c^ BA and CA incubated 2 d and BAana incubated 10 d. Incl. indicates including.

The time to positivity according to culture media was also studied. Regarding the 12 anaerobes detected by classical culture methods, 7 were isolated on plates during the first 3 d of incubation, and 5 were isolated between the 3rd and the 10th day, indicating that 10 d of incubation were still needed for anaerobic solid media. Conversely, for culture media incubated in a CO_2_ atmosphere, the rate of microorganism detection on chocolate agar plates was the same after 2 or 10 d of incubation (44.2 % vs. 43.8 %; p=0.93 ). Concerning the 212 bacteria detected in SF collected in BC bottles, 89.6 % were detected in less than 48 h, and 98.1 % were detected in less than 5 d. Two *Finegoldia* spp. isolates and one *C. acnes* isolate were detected in BC bottles between 5 and 10 d of incubation (also two contaminants: one *Corynebacterium* spp. and one *Kocuria* spp.), and only one microorganism (*Peptostreptococcus anaerobius*) was detected after 10 d.

**Table 4:** Sensitivity and specificity of PCR techniques for the diagnosis of bone and joint infections on synovial fluid.

		16S rDNA	PCR	PCR	PCR	PCR	PCR	PCR	PCR
		PCR	*Staphylococcus*	*S. aureus*	*Streptococcus*	*S. pneumoniae*	*C. acnes*	*B. burgdorferi*	*K. kingae*
Sensitivity (%)									
Anaerobic species, n=25		36.0	–	–	–	–	–	–	–
	incl. *Cutibacterium acnes*, n=12	33.3	–	–	–	–	58.3	–	–
*Streptococcus-Enterococcus* spp., n=47		58.7	–	–	–	–	–	–	–
	incl. *Enterococcus* spp., n=12	33.3	–	–	0	–	–	–	–
	incl. *Streptococcus* spp., n=35	67.6	–	–	91.2	–	–	–	–
	incl. *S. pneumoniae*, n=2	100	–	–	100	100	–	–	–
*Staphylococcus* spp., n=155		47.4	66.2	–	–	–	–	–	–
	incl. *Staphylococcus aureus*, n=88	64.8	81.8	85.2	–	–	–	–	–
	incl. non-*aureus* staphylococci, n=67	24.2	45.5	–	–	–	–	–	–
Gram-negative bacilli, n=44		74.4	–	–	–	–	–	–	–
Other bacteria, n=13		46.2	–	–	–	–	–	–	–
	incl. *Borrelia* spp., n=3	0	–	–	–	–	–	100	–
All bacterial species, n=284		52.3	–	–	–	–	–	–	–
	incl. monomicrobial, n=213	61.9	–	–	–	–	–	–	–
	incl. polymicrobial, n=71	23.9	–	–	–	–	–	–	–
Specificity (%)		90.6	96.7	100.0	98.2	100.0	97.3	100.0	100.0

Incl. indicates including.

### 3.4 Performance of molecular methods vs. culture methods

Of the 284 bacteria responsible for infection in our cohort, 16S rDNA PCR was positive in only 52.3 % of cases (61.9 % of bacteria in monomicrobial samples vs. 23.9 % of bacteria in polymicrobial samples; p<0.001 ). The highest sensitivity for molecular approaches was obtained with specific PCRs (Table 4). Of the 155 staphylococci detected, 16S rDNA PCR was positive in 47.4 % of cases, whereas *Staphylococcus* spp. PCR was positive in 66.2 %. Focusing on *S. aureus* ( n=88 ), 16S rDNA PCR was positive in 64.8 % of cases, *Staphylococcus* spp. PCR was positive in 81.8 % of cases, and *S. aureus*-specific PCR was positive in 85.2 % of cases. The same gain in sensitivity was observed for streptococci  ( n=35 ) which were detected in 67.6 % of cases by 16S rDNA PCR and in 91.2 % by specific PCR. Only two SFs were positive for *S. pneumoniae* PCR, and three were positive for *B. burgdorferi* PCR; no positive PCR was obtained for *K. kingae.* Specificity was 90.6 % for 16S rDNA PCR and between 96.7 % and 100 % for specific PCRs.

### 3.5 Discordance between the different techniques

For 79 out of 266 (29.7 %) bacteria detected in SF, the microorganism was only detected by one of the three techniques compared: conventional culture, BC bottles, or PCR (Table S2). Of these 79 bacteria, 25.3 % concerned polymicrobial samples; 54 (68.4 %) were staphylococci or streptococci. Of the 38 bacteria detected only in culture and not by molecular techniques, 71.1 % ( n=27 ) were staphylococci (including 57.9 % ( n=22 ) coagulase-negative staphylococci). Conversely, 41 bacteria (15.4 %) were detected in SF only by molecular techniques and did not cultivate. Only five (two *Coxiella* and three *Borrelia*) were unable to grow on the culture media used; 23 (56.1 %) were detected from patients having received antibiotics before puncture, and 29 (70.7 %) were detected in non-native joint infection. A total of 17 bacteria were detected only by specific PCR and not by 16S rDNA PCR, including 8 for *Staphylococcus* spp. and *S. aureus* PCRs, 6 for *Streptococcus* spp. PCR, and 3 for *Borrelia burgdorferi* PCR.

### 3.6 Performance of techniques in various subgroups

Among the 242 SFs collected in patients classified as infected, 76 were collected from patients having received antibiotics before puncture, and 90 bacteria were detected. Bacterial detection rates in SF samples collected among patients having received antibiotics and those having not received antibiotics in the 2 weeks before puncture were significantly different with culture techniques ( p<0.05 ) (Table S3) : 26.7 % vs. 53.9 % on the BA plate, 26.7 % vs. 52.3 % on the CA plate at 2 d of incubation, 25.8 % vs. 55.2 % on the BAana plate at 10 d, 36.7 % vs. 58.9 % in SCH broth, 51.1 % vs. 70.0 % in the ANA BC bottle, and 55.6 % vs. 73.3 % in the PED one. Conversely, the bacterial detection rates were not significantly different with PCR techniques: 50.6 % vs. 53.1 % with 16S rDNA PCR ( p=0.70 ), 60.5 % vs. 68.5 % with *Staphylococcus* spp. PCR ( p=0.35 ), 84.2 % vs. 85.5 % with the *S. aureus*-specific PCR ( p=1.0 ), and 83.3 % vs. 95.5 % with the *Streptococcus* spp. PCR ( p=0.28 ).

The various culture and PCR techniques did not show any difference in performance between native joint and non-native joint infections or between acute and chronic infections ( p>0.05 for all techniques; Tables S4 and S5).

### 3.7 Decisional algorithm proposed

According to the data presented herein, a decisional algorithm is proposed to simplify the culture procedures and to define the indications of broad-range and specific PCRs for microbiological diagnosis on SF samples. We propose that SF could be inoculated onto only three solid media (a blood agar plate in aerobic atmosphere, a chocolate blood agar plate in CO_2_ atmosphere incubated for 48 h, and an anaerobic blood agar plate incubated for 10 d) associated with BC bottles incubated for 10 d. When culture is negative with a clinical suspicion of BJI, 16S rDNA, *Staphylococcus* spp.  and *Streptococcus* spp. PCRs could be added. If we apply this algorithm to the cohort presented herein, 93.8 % (227 out of 242) of the positive SFs would have been concluded to be positive for at least one species, and 90.5 % (257 out of 284) of bacteria responsible for BJI would have been detected in SF compared to 93.7 % (266 out of 284) using all the techniques tested on SF in our study.

## 4 Discussion

The present paper reports the first prospective multi-center study that simultaneously explored the performance of culture and molecular bacteriological techniques in order to diagnose BJIs in SF, the results of which led us to propose an algorithm associating culture of SF using three solid media and inoculation into BC bottles, with a total incubation of 10 d, and 16S, *Staphylococcus* spp., and *Streptococcus* spp. PCRs for optimizing bacterial detection.

Compared to conventional culture of SF, BC bottles had a higher detection rate, both for monomicrobial and polymicrobial samples. Previous studies have described the superiority of BC bottles compared to conventional culture media, both with SF and bone samples (Hughes et al., 2011; Bémer et al., 2016; Font-Vizcarra et al., 2010; Peel et al., 2016). Moreover, while BJI diagnosis using conventional agar and broth media requires a prolonged incubation of 2 weeks (Schäfer et al., 2008), the use of BC bottles shortens the time to bacterial detection and reduces the delay in diagnosis compared to enrichment culture broths since almost all positive samples were detected in the first 5 d using BC bottles, as reported in other studies (Hughes et al., 2011; Bémer et al., 2016; Minassian et al., 2014; Peel et al., 2016). This could lead us to propose an incubation time shortened to 10 d when inoculating SF samples in BC bottles. Another interesting point is that inoculation into BC bottles enables an easier monitoring for the laboratory due to the automated detection of bacterial growth and the reduction in technical time for sterile samples, resulting in considerable cost savings, as demonstrated by the Mayo Clinic laboratory (Peel et al., 2017). Of note is that BC flasks used for inoculation of SF must be selected carefully since the various bottles commercially available demonstrate different performances, especially for the detection of *C. acnes* (Jeverica et al., 2020).

However, even if the inoculation in BC bottles shows the highest positivity rate, a better detection of bacteria in SF was observed when associated with conventional culture media. We found that an optimal bacterial detection could be obtained with only three culture media if associated with BC bottles: a blood agar plate and a chocolate agar plate incubated for 48 h and an anaerobic blood agar plate incubated for 10 d. This could allow us to optimize sensitivity while reducing costs and alleviating the technical monitoring of SF samples. Adding conventional culture media to BC bottles is also essential to discriminate between real infection and contaminations, the proportion of culture-positive media being important to take into account for skin commensal bacteria. In 2016, Bémer et al. (2016) proposed a combination of three culture media for BJIs diagnosis: a chocolate agar plate, a Schaedler broth, and a pediatric BC bottle (Bémer et al., 2016). In the present study, results showed that inoculating both a pediatric and an anaerobic BC bottles makes the use of an anaerobic broth unnecessary and that adding a blood agar plate to the chocolate agar plate seems useful for analyzing the various morphologies and hemolytic profiles in the case of infection with various morphotypes.

Regarding molecular assays on SF, several indications can be proposed based on the presented results: suspicion of infection with previous antibiotherapy (Bémer et al., 2014), strong clinical suspicion or biological criteria of BJI with discordant or negative culture results, and suspicion of infection with fastidious or non-cultivable bacteria. In this study, 16S rDNA, *Staphylococcus* spp.  and *Streptococcus* spp. PCRs were the most contributing molecular tests, which is concordant with Levy et al. (2013). The lack of sensitivity of the 16S rRNA PCR followed by Sanger sequencing, as observed by the low detection rate in the present study, has been reported by previous studies (Bémer et al., 2014; Lallemand et al., 2016). This could be linked to the frequently low bacterial inoculums present in BJIs and to the inability of 16S rDNA PCR followed by Sanger sequencing to discriminate between different bacteria in polymicrobial infections (which could be resolved using 16S rDNA PCR followed by whole-genome sequencing). Conversely, a sample positive only in molecular techniques raises the question of whether the bacterium identified is really responsible for an infection as PCR can identify contaminants and DNA from nonviable bacteria. As reported by previous studies (Bémer et al., 2014; Jacquier et al., 2019), the performance of 16S rDNA PCR was good for *S. aureus* and streptococci but also for Gram-negative bacilli; conversely, performance was insufficient for *C. acnes* and coagulase-negative staphylococci. Universal PCR can be helpful, but its indications must be clearly selected, and, above all, clinicians and bacteriologists must be aware of its limits in terms of sensitivity. Specific PCRs are more sensitive but target a single gene found in a single genus or species, thus limiting their use in BJIs where the pathogens are very diverse. Specific PCRs should be prescribed according to the culture results and the clinical context, and an accurate diagnosis may require several specific PCRs to be performed. This means that any remaining volume of SF after culture media inoculation must be frozen to possibly perform PCRs; therefore, synovial fluid volume is sometimes a limiting factor that can prevent the addition of molecular assays. This study was carried out before the development of next-generation sequencing, which could further improve BJI diagnosis, but, currently, few laboratories have access to this new technology due to its cost and complexity.

SF is a very valuable sample for BJI diagnosis as it can be collected at the patient's bedside, it can be directly inoculated into BC bottles by clinicians, and it presents a higher positivity rate in culture compared to tissues and bone samples (Bémer et al., 2016). However, it also presents an important drawback for bacteriological diagnosis: SF is sometimes collected alone, without bone samples, and, in this case, the interpretation of positive bacteriological results for a potential contaminant may be difficult. The use of several diagnostic techniques (culture, blood culture bottles, PCRs) can help differentiate between contaminant and infection, depending on the number of positive techniques for the same microorganism.

## 5 Conclusions

The present study found that a combination of culture and molecular methods on SF allows the optimization of bacterial detection. This combination of techniques would allow us to simplify the bacteriological process and lead to reductions in associated costs.

## Supplement

10.5194/jbji-9-37-2024-supplement The supplement related to this article is available online at: https://doi.org/10.5194/jbji-9-37-2024-supplement.

## Data Availability

All relevant materials and data supporting the findings of this study are contained within the paper. More detailed data are available from the corresponding author upon reasonable request.

## Appendix

The supplement related to this article is available online at: https://doi.org/10.5194/jbji-9-37-2024-supplement.

## Team list

Members of the PIRLA investigator group included infectious disease specialists, surgeons, rheumatologists, and microbiologists from different sites in Lyon and Saint-Étienne, France. List of members: Frédéric Laurent (coordinator, Hospices Civils de Lyon, Lyon, France), Caroline Debette, Tristan Ferry, Sébastien Lustig, Anya Madelaine, Tanguy Mouton, Philippe Neyret, Olivier Reynaud, Elvire Servien, Franck Trouillet, Vincent Villa (Hospices Civils de Lyon, Lyon, France), Adamah Amouzougan, Elisabeth Botelho-Nevers, Karima Boussoualim, Bertrand Boyer, Céline Cazorla, Frédéric Farizon, Anne Fresard, Amandine Gagneux-Brunon, Giorgio Gresta, Frédéric Lucht, Marie France Lutz, Hubert Marotte, Thomas Neri, Remy Philippot, Florence Suy, Thierry Thomas (CHU de Saint-Étienne, Saint-Étienne, France), Dominique Avet, Laurent Beguin, Olivier Carnesecchi, Francois Chalencon, Florent Delangle, Francois-Xavier Verdot (Centre Hospitalier Privé de la Loire, Saint-Étienne, France), Laurent Dupre Latour, Loic Schneider (Clinique Mutualiste de la Loire, Saint-Étienne, France).

## References

[bib1.bib1] Bauer HM, Ting Y, Greer CE, Chambers JC, Tashiro CJ, Chimera J, Reingold A, Manos MM (1991). Genital human papillomavirus infection in female university students as determined by a PCR-based method. JAMA.

[bib1.bib2] Bémer P, Plouzeau C, Tande D, Léger J, Giraudeau B, Valentin AS, Jolivet-Gougeon A, Vincent P, Corvec S, Gibaud S, Juvin ME, Héry-Arnaud G, Lemarié C, Kempf M, Bret L, Quentin R, Coffre C, de Pinieux G, Bernard L, Burucoa C, Centre de Référence des Infections Ostéo-articulaires du Grand Ouest (CRIOGO) Study Team (2014). Evaluation of 16S rRNA gene PCR sensitivity and specificity for diagnosis of prosthetic joint infection: a prospective multicenter cross-sectional study. J Clin Microbiol.

[bib1.bib3] Bémer P, Léger J, Tandé D, Plouzeau C, Valentin AS, Jolivet-Gougeon A, Lemarié C, Kempf M, Héry-Arnaud G, Bret L, Juvin ME, Giraudeau B, Corvec S, Burucoa C, Centre de Référence des Infections Ostéo-articulaires du Grand Ouest (CRIOGO) Study Team (2016). How many samples and how many culture media to diagnose a prosthetic joint infection: a clinical and microbiological prospective multicenter study. J Clin Microbiol.

[bib1.bib4] Carvalho MdaGS, Tondella ML, McCaustland K, Weidlich L, McGee L, Mayer LW, Steigerwalt A, Whaley M, Facklam RR, Fields B, Carlone G, Ades EW, Dagan R, Sampson JS (2007). Evaluation and improvement of real-time PCR assays targeting *lytA*,
*ply*, and *psaA* genes for detection of pneumococcal DNA. J Clin Microbiol.

[bib1.bib5] Cherkaoui A, Ceroni D, Emonet S, Lefevre Y, Schrenzel J (2009). Molecular diagnosis of *Kingella kingae* osteoarticular infections by specific real-time PCR assay. J Med Microbiol.

[bib1.bib6] Chometon S, Benito Y, Chaker M, Boisset S, Ploton C, Bérard J, Vandenesch F, Freydiere AM (2007). Specific real-time polymerase chain reaction places *Kingella kingae* as the most common cause of osteoarticular infections in young children. Pediatr Infect Dis J.

[bib1.bib7] Font-Vizcarra L, García S, Martínez-Pastor JC, Sierra JM, Soriano A (2010). Blood culture flasks for culturing synovial fluid in prosthetic joint infections. Clin Orthop Relat Res.

[bib1.bib8] Harris KA, Hartley JC (2003). Development of broad-range 16S rDNA PCR for use in the routine diagnostic clinical microbiology service. J Med Microbiol.

[bib1.bib9] Hughes HC, Newnham R, Athanasou N, Atkins BL, Bejon P, Bowler ICJW (2011). Microbiological diagnosis of prosthetic joint infections: a prospective evaluation of four bacterial culture media in the routine laboratory. Clin Microbiol Infect.

[bib1.bib10] Jacquier H, Fihman V, Amarsy R, Vicaut E, Bousson V, Cambau E, Crémieux A-C, Delcey V, Hannouche D, Kaci R, Laredo J-D, Meunier F, Nizard R, Ottaviani S, Parlier C, Richette P, Sellier P, Zadegan F, Lioté F, Berçot B (2019). Benefits of polymerase chain reaction combined with culture for the diagnosis of bone and joint infections: a prospective test performance study. Open Forum Infect Dis.

[bib1.bib11] Jeverica S, El Sayed F, Čamernik P, Kocjančič B, Sluga B, Rottman M, Papst L (2020). Growth detection of *Cutibacterium acnes* from orthopaedic implant-associated infections in anaerobic bottles from BACTEC and BacT/ALERT blood culture systems and comparison with conventional culture media. Anaerobe.

[bib1.bib12] Lallemand E, Coiffier G, Arvieux C, Brillet E, Guggenbuhl P, Jolivet-Gougeon A (2016). MALDI-TOF MS performance compared to direct examination, culture, and 16S rDNA PCR for the rapid diagnosis of bone and joint infections. Eur J Clin Microbiol Infect Dis.

[bib1.bib13] Larsen LH, Lange J, Xu Y, Schønheyder HC (2012). Optimizing culture methods for diagnosis of prosthetic joint infections: a summary of modifications and improvements reported since 1995. J Med Microbiol.

[bib1.bib14] Levy P-Y, Fournier P-E, Fenollar F, Raoult D (2013). Systematic PCR detection in culture-negative osteoarticular infections. Am J Med.

[bib1.bib15] Lew DP, Waldvogel FA (2004). Osteomyelitis. Lancet.

[bib1.bib16] Minassian AM, Newnham R, Kalimeris E, Bejon P, Atkins BL, Bowler ICJW (2014). Use of an automated blood culture system (BD BACTEC™) for diagnosis of prosthetic joint infections: easy and fast. BMC Infect Dis.

[bib1.bib17] Osmon DR, Berbari EF, Berendt AR, Lew D, Zimmerli W, Steckelberg JM, Rao N, Hanssen A, Wilson WR, Infectious Diseases Society of America (2013). Diagnosis and management of prosthetic joint infection: clinical practice guidelines by the Infectious Diseases Society of America. Clin Infect Dis.

[bib1.bib18] Paule SM, Pasquariello AC, Hacek DM, Fisher AG, Thomson RB, Kaul KL, Peterson LR (2004). Direct detection of *Staphylococcus aureus* from adult and neonate nasal swab specimens using real-time polymerase chain reaction. J Mol Diagn.

[bib1.bib19] Peel TN, Dylla BL, Hughes JG, Lynch DT, Greenwood-Quaintance KE, Cheng AC, Mandrekar JN, Patel R (2016). Improved diagnosis of prosthetic joint infection by culturing periprosthetic tissue specimens in blood culture bottles. MBio.

[bib1.bib20] Peel TN, Sedarski JA, Dylla BL, Shannon SK, Amirahmadi F, Hughes JG, Cheng AC, Patel R (2017). Laboratory workflow analysis of culture of periprosthetic tissues in blood culture bottles. J Clin Microbiol.

[bib1.bib21] Relman D (1993). Diagnostic molecular microbiology Principles and applications.

[bib1.bib22] Schäfer P, Fink B, Sandow D, Margull A, Berger I, Frommelt L (2008). Prolonged bacterial culture to identify late periprosthetic joint infection: a promising strategy. Clin Infect Dis.

[bib1.bib23] Sfanos KS, Isaacs WB (2008). An evaluation of PCR primer sets used for detection of *Propionibacterium acnes* in prostate tissue samples. Prostate.

[bib1.bib24] Tande AJ, Patel R (2014). Prosthetic joint infection. Clin Microbiol Rev.

