# Supplementary material for: Optimized decision algorithm for the microbiological diagnosis of osteoarticular infections in adults using synovial fluid samples: a prospective study in two French hospitals including 423 samples of synovial fluid
Source: J Bone Jt Infect. 2024 Feb 6;9(1):37–48. doi: 10.5194/jbji-9-37-2024 (PMC11004666; doi:10.5194/jbji-9-37-2024)
Supplement: The supplement related to this article is available online at: https://doi.org/10.5194/jbji-9-37-2024-supplement. [file jbji-9-37-supplement.pdf]

Supplement of J. Bone Joint Infect., 9, 37–48, 2024
https://doi.org/10.5194/jbji-9-37-2024-supplement
© Author(s) 2024. CC BY 4.0 License.

JBJI Journal of Bone and Joint Infection Open Access

Supplement of

Optimized decision algorithm for the microbiological diagnosis of osteoarticular infections in adults using synovial fluid samples: a prospective study in two French hospitals including 423 samples of synovial fluid

Céline Dupieux et al.

Correspondence to: Céline Dupieux (celine.dupieux@chu-lyon.fr)

The copyright of individual parts of the supplement might differ from the article licence.

S1: Interpretation of bacteriological results of osteoarticular samples according to the French 2022 national recommendations.

English translation of the Référentiel en Microbiologie médicale, REMIC, 7th edition, chapter 32, p377:

"The interpretation of microbiological results in the context of bone and joint infections (BJIs) is complex: it must take into account the clinical context (acute *vs*. chronic infection, osteomyelitis *vs*. native joint arthritis *vs*. prosthetic joint infection, prior antibiotic therapy, for example), the species identified, the nature and number of positive samples and, possibly for the latter, the number of positive media and colonies observed.

This interpretation is generally based on the study of several deep samples from the same patient, but there is no definitive consensus on the exact microbiological criteria for diagnosing BJI. The likelihood of infection increases with the number of positive samples for the same bacterium. In the case of samples collected from prosthetic joints, some studies report that the probability is over 95% when at least three samples are positive, around 20% when two samples are positive, only 10% when a single sample is positive, and 3% when cultures are negative.

There are currently several microbiological definitions of the periprosthetic joint infection. In all cases, if two intraoperative samples are positive for the same microorganism (defined by identification and antibiotic susceptibility profile), the infection is microbiologically defined. In the absence of this criterion, some consider the following elements:

- The presence of two deep samples collected at different times (1 intraoperative sample + 1 sample from a joint puncture or blood culture) and positive for the same bacterium;
- The presence of a single deep sample positive for a bacterium that does not belong to the skin flora and for which the question of contamination does not arise (*Staphylococcus aureus*, Enterobacteriaceae, *Pseudomonas aeruginosa*, *Streptococcus pneumoniae*, *Salmonella*, *Listeria*, *Neisseria gonorrhoeae*, *Campylobacter*, *Pasteurella*, for example) [IDSA criterion];
- The presence of a single positive sample in the context of an infection (elevated inflammatory markers, evocative joint cytology, etc.) [MSIS criterion].

In other cases, confirmation of BJI requires analysis of all the clinical and paraclinical data in the file and a dialogue between microbiologists and clinicians.

In the case of septic arthritis, as a single sample is collected (synovial fluid), confirmation of the pathogenic or contaminating nature of any bacteria isolated can only be based on a combination of the cytology, the nature of the bacterial species isolated, the number of positive media and the number of colonies on solid media."

Table S1: Contaminant bacteria obtained from 423 joint fluids using cultural and molecular approaches.

		Number of bacteria detected							
		Culture			BC bottles		PCR		
	n	2-3 days only	10 days only	2-3 and 10 days	1 bottle	2 bottles	16S rDNA PCR only	Specific PCR only	16S rDNA and specific PCR
Anaerobic species	17	0	5	0	1	0	0	11	0
incl. *Cutibacterium acnes*	16	0	5	0	0	0	0	11	0
incl. *Cutibacterium granulosum*	1	0	0	0	1	0	0	NA	NA
Streptococcus spp	7	0	0	1	0	0	0	4	2
Staphylococcus spp	24	2	1	1	8	1	0	11	0
incl. CoNS except *S. epidermidis*	12	2	1	0	3	0	0	6	0
incl. *S. epidermidis*	12	0	0	1	5	1	0	5	0
Gram-negative bacilli	2	0	0	1	0	0	1	NA	NA
incl. *Haemophilus* spp	2	0	0	1	0	0	1	0	0
Other bacteria	8	0	1	0	2	0	5	NA	NA
incl. *Bacillus* spp	1	0	1	0	0	0	0	NA	NA
incl. *Kocuria* spp	1	0	0	0	1	0	0	NA	NA
incl. *Corynebacterium* spp	3	0	0	0	1	0	2	NA	NA
incl. *Nocardioides* spp	1	0	0	0	0	0	1	NA	NA
incl. *Lactococcus* spp	1	0	0	0	0	0	1	NA	NA
incl. *Paracoccus* spp	1	0	0	0	0	0	1	NA	NA
All bacterial species	58	2	7	3	11	1	6	26	2

Table S2: Details of bacteria responsible for infection and detected by only one technique (classical culture, BC bottles, or PCR techniques) in synovial fluid.

	Number of bacteria detected			
	Classical culture only	BC bottles only	PCR only	Total
Bacteria				
Cutibacterium acnes	0	1	2	3
Anaerobic species except *C. acnes*	1	1	0	2
Streptococcus spp	0	1	13	14
Enterococcus spp	1	2	1	4
S. aureus	1	4	8	13
CoNS	3	19	5	27
Gram-negative bacilli	0	3	6	9
Other bacteria	1	0	6	7
Total	7	31	41	79
Context				
Polymicrobial infection, n (%)	6 (86)	12 (39)	2 (5)	20 (25)
Chronic infection, n (%)	6 (86)	13 (42)	22 (54)	41 (52)
Previous antibiotics (15 days), n (%)	4 (57)	13 (42)	23 (56)	40 (51)
Non-native infection, n (%)	4 (57)	20 (65)	29 (71)	53 (67)

Table S3: Sensitivity of each culture medium and PCR evaluated for the detection of bacteria responsible for 242 bone and joint infections in synovial fluid in patients having received antibiotics during the 15 days before SF *vs.* the patients having not received antibiotics.

		CULTURE								PCR						
	Infections (n)	BA aero. (2 days)	CA CO_2 (2 days)	BA ana. (3 days)	CA CO_2 (10 days)	BA ana. (10 days)	SCH broth (10 days)	BC ANA	BC PED	16S rDNA PCR	PCR *Staphylococcus*	PCR *S. aureus*	PCR *Streptococcus*	PCR *S. pneumoniae*	PCR *C. acnes*	PCR *B. burgdorferi*
**Anaerobic species**	**25**	**0.0**	**0.0**	**28.0**	**0.0**	**40.0**	**40.0**	**48.0**	**12.0**	**36.0**						
antibiotics	8	0.0	0.0	50.0	0.0	62.5	75.0	50.0	12.5	37.5						
no antibiotics	17	0.0	0.0	17.6	0.0	29.4	23.5	47.1	11.8	35.3						
**Anaerobes except *C. acnes***	**13**	**0.0**	**0.0**	**38.5**	**0.0**	**46.2**	**53.8**	**53.8**	**7.7**	**38.5**						
antibiotics	8	0.0	0.0	50.0	0.0	62.5	75.0	50.0	12.5	37.5						
no antibiotics	5	0.0	0.0	20.0	0.0	20.0	20.0	60.0	0.0	40.0						
Cutibacterium acnes	**12**	**0.0**	**0.0**	**16.7**	**0.0**	**33.3**	**25.0**	**41.7**	**16.7**	**33.3**					**58.3**	
antibiotics	0															
no antibiotics	12	0.0	0.0	16.7	0.0	33.3	25.0	41.7	16.7	33.3					58.3	
***Streptococcus-Enterococcus* spp**	**47**	**38.3**	**36.2**	**38.3**	**36.2**	**40.4**	**40.4**	**54.3**	**54.3**	**58.7**						
antibiotics	17	35.3	29.4	29.4	29.4	29.4	35.3	52.9	58.8	58.8						
no antibiotics	30	40.0	40.0	43.3	40.0	46.7	43.3	55.2	51.7	58.6						
***Enterococcus* spp**	**12**	**41.7**	**41.7**	**41.7**	**41.7**	**50.0**	**58.3**	**66.7**	**58.3**	**33.3**						
antibiotics	5	60.0	60.0	60.0	60.0	60.0	80.0	60.0	60.0	20.0						
no antibiotics	7	28.6	28.6	28.6	28.6	42.9	42.9	71.4	57.1	42.9						
***Streptococcus* spp**	**35**	**37.1**	**34.3**	**37.1**	**34.3**	**37.1**	**34.3**	**50.0**	**52.9**	**67.6**			**91.2**			
antibiotics	12	25.0	16.7	16.7	16.7	16.7	16.7	50.0	58.3	75.0			83.3			
no antibiotics	23	43.5	43.5	47.8	43.5	47.8	43.5	50.0	50.0	63.6			95.5			
S. pneumoniae	**2**	**50.0**	**50.0**	**50.0**	**50.0**	**50.0**	**50.0**	**50.0**	**50.0**	**100.0**			**100.0**	**100.0**		
antibiotics	0															
no antibiotics	2	50.0	50.0	50.0	50.0	50.0	50.0	50.0	50.0	100.0			100.0	100.0		
***Staphylococcus* spp.**	**155**	**54.2**	**54.2**	**51.3**	**53.9**	**54.5**	**58.7**	**73.7**	**79.7**	**47.4**	**66.2**					
antibiotics	44	22.7	25.0	20.9	25.0	20.5	31.8	54.5	59.1	34.9	60.5					
no antibiotics	111	66.7	65.8	63.3	65.5	68.2	69.4	81.5	88.1	52.3	68.5					
S. aureus	**88**	**76.1**	**77.3**	**75.9**	**77.3**	**75.0**	**77.3**	**75.6**	**85.2**	**64.8**	**81.8**	**85.2**				
antibiotics	19	26.3	26.3	22.2	31.6	21.1	31.6	42.1	52.6	52.6	78.9	84.2				
no antibiotics	69	89.9	91.3	89.9	89.9	89.9	89.9	85.1	94.2	68.1	82.6	85.5				
**Non-*aureus* staphylococci**	**67**	**25.4**	**23.9**	**18.5**	**22.7**	**27.3**	**34.3**	**71.2**	**72.3**	**24.2**	**45.5**					
antibiotics	25	20.0	24.0	20.0	20.0	20.0	32.0	64.0	64.0	20.8	45.8					
no antibiotics	42	28.6	23.8	17.5	24.4	31.7	35.7	75.6	77.5	26.2	45.2					
**Gram-negative bacilli**	**44**	**58.1**	**46.5**	**31.7**	**45.2**	**38.1**	**60.5**	**63.6**	**81.8**	**74.4**						
antibiotics	15	53.3	40.0	21.4	35.7	28.6	46.7	53.3	73.3	93.3						
no antibiotics	29	60.7	50.0	37.0	50.0	42.9	67.9	69.0	86.2	64.3						
**Other bacteria**	**13**	**7.7**	**30.8**	**0.0**	**30.8**	**0.0**	**0.0**	**15.4**	**30.8**	**46.2**						**100.0**
antibiotics	6	0.0	33.3	0.0	50.0	0.0	0.0	16.7	33.3	50.0						100.0
no antibiotics	7	14.3	28.6	0.0	14.3	0.0	0.0	14.3	28.6	42.9						
**All bacterial species**	**284**	**45.2**	**44.2**	**41.7**	**43.8**	**45.9**	**51.8**	**63.9**	**67.6**	**52.3**						
antibiotics	90	26.7	26.7	23.9	27.0	25.8	36.7	51.1	55.6	50.6						
no antibiotics	194	53.9	52.3	50.0	51.6	55.2	58.9	70.0	73.3	53.1						

Table S4: Sensitivity of each culture medium and PCR evaluated for the detection of bacteria responsible for 242 bone and joint infections in synovial fluid in native joint infections *vs.* non-native joint infections.

		CULTURE								PCR					
	Infections (n)	BA aero. (2 days)	CA CO_2 (2 days)	BA ana. (3 days)	CA CO_2 (10 days)	BA ana. (10 days)	SCH broth (10 days)	BC ANA	BC PED	16S rDNA PCR	*Staphylococcus*	PCR *S. aureus*	PCR *Streptococcus*	PCR *S. pneumoniae*	PCR *C. acnes*
**Anaerobic species**	**25**	**0.0**	**0.0**	**28.0**	**0.0**	**40.0**	**40.0**	**48.0**	**12.0**	**36.0**					
native joint	3	0.0	0.0	0.0	0.0	0.0	0.0	33.3	0.0	0.0					
non-native joint	22	0.0	0.0	31.8	0.0	45.5	45.5	50.0	13.6	40.9					
**Anaerobes except *C. acnes***	**13**	**0.0**	**0.0**	**38.5**	**0.0**	**46.2**	**53.8**	**53.8**	**7.7**	**38.5**					
native joint	0														
non-native joint	13	0.0	0.0	38.5	0.0	46.2	53.8	53.8	7.7	38.5					
Cutibacterium acnes	**12**	**0.0**	**0.0**	**16.7**	**0.0**	**33.3**	**25.0**	**41.7**	**16.7**	**33.3**					**58.3**
native joint	3	0.0	0.0	0.0	0.0	0.0	0.0	33.3	0.0	0.0					0.0
non-native joint	9	0.0	0.0	22.2	0.0	44.4	33.3	44.4	22.2	44.4					77.8
***Streptococcus-Enterococcus* spp**	**47**	**38.3**	**36.2**	**38.3**	**36.2**	**40.4**	**40.4**	**54.3**	**54.3**	**58.7**					
native joint	18	35.7	35.7	35.7	35.7	40.0	37.5	68.8	66.7	78.6					
non-native joint	29	50.0	46.2	50.0	46.2	50.0	50.0	53.8	57.7	66.7					
***Enterococcus* spp**	**12**	**41.7**	**41.7**	**41.7**	**41.7**	**50.0**	**58.3**	**66.7**	**58.3**	**33.3**					
native joint	5	20.0	20.0	20.0	20.0	40.0	60.0	60.0	40.0	40.0					
non-native joint	7	57.1	57.1	57.1	57.1	57.1	57.1	71.4	71.4	28.6					
***Streptococcus* spp**	**35**	**37.1**	**34.3**	**37.1**	**34.3**	**37.1**	**34.3**	**50.0**	**52.9**	**67.6**			**91.2**		
native joint	13	30.8	30.8	30.8	30.8	30.8	23.1	61.5	61.5	75.0			91.7		
non-native joint	22	40.9	36.4	40.9	36.4	40.9	40.9	42.9	47.6	63.6			90.9		
S. pneumoniae	**2**	**50.0**	**50.0**	**50.0**	**50.0**	**50.0**	**50.0**	**50.0**	**50.0**	**100.0**			**100.0**	**100.0**	
native joint	2	50.0	50.0	50.0	50.0	50.0	50.0	50.0	50.0	100.0			100.0	100.0	
non-native joint	0														
***Staphylococcus* spp.**	**155**	**54.2**	**54.2**	**51.3**	**53.9**	**54.5**	**58.7**	**73.7**	**79.2**	**47.4**	**66.2**				
native joint	48	47.9	50.0	47.8	46.8	48.9	54.2	70.2	75.0	45.8	64.6				
non-native joint	107	57.0	56.1	52.8	57.0	57.0	60.7	75.2	81.1	48.1	67.0				
S. aureus	**88**	**76.1**	**77.3**	**75.9**	**77.3**	**75.0**	**77.3**	**75.6**	**85.2**	**64.8**	**81.8**	**85.2**			
native joint	27	74.1	77.8	74.1	74.1	74.1	74.1	70.4	85.2	63.0	74.1	77.8			
non-native joint	61	77.0	77.0	76.7	78.7	75.4	78.7	78.0	85.2	65.6	85.2	88.5			
**Non-*aureus* staphylococci**	**67**	**25.4**	**23.9**	**18.5**	**22.7**	**27.3**	**34.3**	**71.2**	**71.2**	**24.2**	**45.5**				
native joint	21	14.3	14.3	10.5	10.0	15.0	28.6	70.0	61.9	23.8	52.4				
non-native joint	46	30.4	28.3	21.7	28.3	32.6	37.0	71.7	75.6	24.4	42.2				
**Gram-negative bacilli**	**44**	**58.1**	**46.5**	**31.7**	**45.2**	**38.1**	**60.5**	**63.6**	**81.8**	**74.4**					
native joint	13	69.2	53.8	18.2	50.0	33.3	61.5	69.2	92.3	61.5					
non-native joint	31	53.3	43.3	36.7	43.3	40.0	60.0	61.3	77.4	80.0					
**Other bacteria**	**13**	**7.7**	**30.8**	**0.0**	**30.8**	**0.0**	**0.0**	**15.4**	**30.8**	**46.2**					
native joint	6	0.0	33.3	0.0	33.3	0.0	0.0	16.7	33.3	16.7					
non-native joint	7	14.3	28.6	0.0	28.6	0.0	0.0	14.3	28.6	71.4					
**All bacterial species**	**284**	**45.2**	**44.2**	**41.7**	**43.8**	**45.9**	**51.8**	**63.9**	**67.4**	**52.3**					
native joint	88	44.0	45.2	36.3	42.7	39.8	47.1	64.7	70.6	50.0					
non-native joint	196	47.4	45.3	45.5	45.8	50.0	55.2	64.9	67.7	55.6					

Table S5: Sensitivity of each culture medium and PCR evaluated for the detection of bacteria responsible for 242 bone and joint infections in synovial fluid in acute *vs.* chronic infections.

		CULTURE								PCR					
	Infections (n)	BA aero. (2 days)	CA CO_2 (2 days)	BA ana. (3 days)	CA CO_2 (10 days)	BA ana. (10 days)	SCH broth (10 days)	BC ANA	BC PED	16S rDNA PCR	*Staphylococcus*	PCR *S. aureus*	PCR *Streptococcus*	PCR *S. pneumoniae*	PCR *C. acnes*
**Anaerobic species**	**25**	**0.0**	**0.0**	**28.0**	**0.0**	**40.0**	**40.0**	**48.0**	**12.0**	**36.0**					
acute infection	3 *	0.0	0.0	33.3	0.0	33.3	33.3	66.7	0.0	33.3					
chronic infection	21 *	0.0	0.0	23.8	0.0	38.1	42.9	42.9	14.3	33.3					
**Anaerobes except *C. acnes***	**13**	**0.0**	**0.0**	**38.5**	**0.0**	**46.2**	**53.8**	**53.8**	**7.7**	**38.5**					
acute infection	0 *														
chronic infection	12 *	0.0	0.0	33.3	0.0	41.7	58.3	50.0	8.3	33.3					
Cutibacterium acnes	**12**	**0.0**	**0.0**	**16.7**	**0.0**	**33.3**	**25.0**	**41.7**	**16.7**	**33.3**					**58.3**
acute infection	3	0.0	0.0	33.3	0.0	33.3	33.3	66.7	0.0	33.3					66.7
chronic infection	9	0.0	0.0	11.1	0.0	33.3	22.2	33.3	22.2	33.3					55.6
***Streptococcus-Enterococcus* spp**	**47**	**38.3**	**36.2**	**38.3**	**36.2**	**40.4**	**40.4**	**54.3**	**54.3**	**58.7**					
acute infection	27	25.9	25.9	29.6	25.9	33.3	29.6	57.7	53.8	65.4					
chronic infection	20	55.0	50.0	50.0	50.0	50.0	55.0	50.0	55.0	50.0					
***Enterococcus* spp**	**12**	**41.7**	**41.7**	**41.7**	**41.7**	**50.0**	**58.3**	**66.7**	**58.3**	**33.3**					
acute infection	5	20.0	20.0	20.0	20.0	40.0	40.0	80.0	60.0	40.0					
chronic infection	7	57.1	57.1	57.1	57.1	57.1	71.4	57.1	57.1	28.6					
***Streptococcus* spp**	**35**	**37.1**	**34.3**	**37.1**	**34.3**	**37.1**	**34.3**	**50.0**	**52.9**	**67.6**			**91.2**		
acute infection	22	27.3	27.3	31.8	27.3	31.8	27.3	52.4	52.4	71.4			95.2		
chronic infection	13	53.8	46.2	46.2	46.2	46.2	46.2	46.2	53.8	61.5			84.6		
S. pneumoniae	**2**	**50.0**	**50.0**	**50.0**	**50.0**	**50.0**	**50.0**	**50.0**	**50.0**	**100.0**			**100.0**	**100.0**	
acute infection	2	50.0	50.0	50.0	50.0	50.0	50.0	50.0	50.0	100.0			100.0	100.0	
chronic infection	0														
***Staphylococcus* spp.**	**155**	**54.2**	**54.2**	**51.3**	**53.9**	**54.5**	**58.7**	**73.7**	**79.7**	**47.4**	**66.2**				
acute infection	73	56.2	56.2	53.5	53.4	54.8	63.0	75.3	80.8	50.0	63.9				
chronic infection	82	52.4	52.4	49.4	54.3	54.3	54.9	72.2	78.8	45.1	68.3				
S. aureus	**88**	**76.1**	**77.3**	**75.9**	**77.3**	**75.0**	**77.3**	**75.6**	**85.2**	**64.8**	**81.8**	**85.2**			
acute infection	43	81.4	83.7	81.0	81.4	79.1	81.4	79.1	88.4	74.4	81.4	83.7			
chronic infection	45	71.1	71.1	71.1	73.3	71.1	73.3	72.1	82.2	55.6	82.2	86.7			
**Non-*aureus* staphylococci**	**67**	**25.4**	**23.9**	**18.5**	**22.7**	**27.3**	**34.3**	**71.2**	**72.3**	**24.2**	**45.5**				
acute infection	30	20.0	16.7	13.8	13.3	20.0	36.7	70.0	70.0	13.8	37.9				
chronic infection	37	29.7	29.7	22.2	30.6	33.3	32.4	72.2	74.3	32.4	51.4				
**Gram-negative bacilli**	**44**	**58.1**	**46.5**	**31.7**	**45.2**	**38.1**	**60.5**	**63.6**	**81.8**	**74.4**					
acute infection	20	63.2	52.6	33.3	52.6	42.1	57.9	65.0	80.0	73.7					
chronic infection	24	54.2	41.7	30.4	39.1	34.8	62.5	62.5	83.3	75.0					
**Other bacteria**	**13**	**7.7**	**30.8**	**0.0**	**30.8**	**0.0**	**0.0**	**15.4**	**30.8**	**46.2**					
acute infection	6	0.0	33.3	0.0	33.3	0.0	0.0	16.7	33.3	33.3					
chronic infection	7	14.3	28.6	0.0	28.6	0.0	0.0	14.3	28.6	57.1					
**All bacterial species**	**284**	**45.2**	**44.2**	**41.7**	**43.8**	**45.9**	**51.8**	**63.9**	**67.6**	**52.3**					
acute infection	129 *	46.9	46.9	42.4	45.3	45.3	52.0	67.2	71.1	55.6					
chronic infection	154 *	44.2	42.2	40.8	42.8	46.1	51.9	60.9	65.1	49.4					

* In 1 case, no information was available on whether the infection was acute or chronic.
